# Two-stage (photoautotrophy and heterotrophy) cultivation enables efficient production of bioplastic poly-3-hydroxybutyrate in auto-sedimenting cyanobacterium

**DOI:** 10.1038/srep37121

**Published:** 2016-11-15

**Authors:** Tanakarn Monshupanee, Palida Nimdach, Aran Incharoensakdi

**Affiliations:** 1 Department of Biochemistry, Faculty of Science, Chulalongkorn University, Bangkok 10330, Thailand

## Abstract

Sustainable production of bioplastics by heterotrophic microbes has been restricted by the limited resources of organic substrates and the energy required for biomass harvest. Here, the easy-to-harvest cyanobacterium (*Chlorogloea fritschii* TISTR 8527), from which the biomass instantaneously settled to the bottom of liquid culture, was utilized to produce poly-3-hydroxybutyrate (PHB) using a two-stage cultivation strategy. The cells were first pre-grown under normal photoautotrophy to increase their biomass and then recultivated under a heterotrophic condition with a single organic substrate to produce the product. Through optimization of this two-stage cultivation, the mass conversion efficiency of acetate substrate to PHB was obtained at 51 ± 7% (w/w), the comparable level to the theoretical biochemical conversion efficiency of acetate to PHB. This two-stage cultivation that efficiently converted the substrate to the product, concurrent with a reduced culture biomass, may be applicable for the production of other biopolymers by cyanobacteria.

Microbial production of biodegradable plastics by heterotrophy is an effective method due to its superior product versatility and productivity compared to those obtained by photoautotrophy. However, such heterotrophic systems, which have mainly been established in bacteria, rely on a substantial consumption of composite organic compounds. Thus, a heterotrophic approach that requires a lower amount of a simple organic substrate is more desirable. Heterotrophic approaches have been described before in the two-stage cultivation of cyanobacteria, where the cells were pre-grown under photoautotrophy to increase their biomass, and subsequently cultured under heterotrophy with a single type of organic substrate. These two-stage cultivation systems have been reported in a number of cyanobacteria for the production of the biodegradable plastic poly-3-hydroxybutyrate (PHB)^1^,^2^,^3^, glycogen (GL)^3^,^4^,^5^ or lipids (LP)^3^,^6^. However, the conversion efficiency (*CE*) of the organic substrate to the products has not been examined in these cyanobacteria, despite the relatively low concentration (0.05–3% w/v) of the organic substrate used in the production process^1^,^2^,^3^,^4^,^5^,^6^.

The theoretical value of the maximum cellular conversion efficiency (*CE*) of glucose or acetate to PHB is 48% (w/w)^7^. Although a *CE* of glucose to PHB of 36–52% (w/w) has been described in bacteria, the cells also required other types of composite organic substrates, such as yeast extract and/or peptone, for the PHB production^8^,^9^,^10^. Thus, the definite determination of the *CE* from a specific organic substrate to a product was restricted by the presence of the composite organic compounds in these heterotrophic cultivations, particularly in bacterial and yeast systems. In cyanobacteria, a more definitive *CE* determination of a specific organic substrate to a product is possible by utilizing the two-stage culture system, where the cells are first grown under photoautotrophy, followed by heterotrophic cultivation with only a specific organic substrate.

Biomass harvest has been a major obstacle for producing bioplastic and non-excreting molecules by cyanobacteria^11^,^12^. Centrifugation, filtration and chemical flocculation have all been used for cell harvesting^12^,^13^,^14^, but these techniques suffer from a required extended time, cost and energy^14^ as well as a reduction in the economic and environmental viability of the method. Therefore, a strategy utilizing auto-sedimenting biomass with no requirement for extra energy and a reduced cost and time for cell harvesting will be a promising advantage.

To efficiently produce PHB by cyanobacteria, three key features are needed to be developed: (i) an effective cell harvesting method, (ii) a strain yielding a high biomass level and (iii) a cultivation strategy that efficiently converts the organic substrate to PHB. To establish such a system in cyanobacteria, the easy-to-harvest cyanobacterium (*Chlorogloea fritschii*) that has a living biomass capable of spontaneous sedimentation was selected. The cells were evaluated for their biomass yield and accumulation levels of PHB under cultivation with different nutrients, organic substrates and light/dark conditions. Subsequently, two-stage cultivation of the cells under photoautotrophy followed by heterotrophy with a single organic substrate was performed, and the *CE* of the organic substrate to PHB was determined.

## Results

### Auto-sedimentation of *Chlorogloea fritschii* TISTR 8527

**Figure 1:**
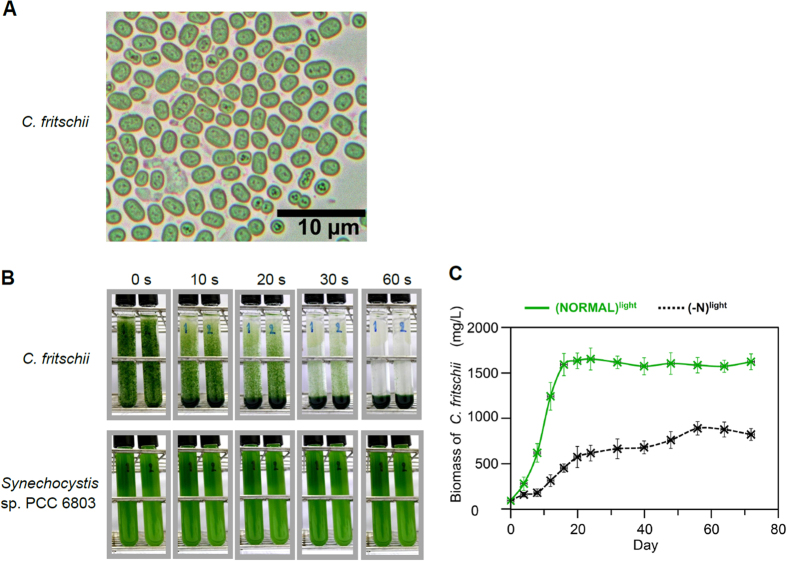
Auto-sedimentation and cell growth of *C. fritschii*. (**A**) Morphology of *C. fritschii* cell clusters under the light microscope (1000x magnification). (**B**) Auto-sedimentation of the 16-d old photoautotrophic cultures. Fifteen ml of cultures (duplicate tubes: 1 and 2) were transferred to glass tubes (10-cm height) and left under natural gravity. (**C**) Photoautotrophic growth of *C. fritschii* under the normal nutrient condition (NORMAL) or nitrogen limitation (-N). Values are the average ±1 SD of four independent cultures.

We previously screened 137 cyanobacterial strains for their PHB accumulation ability^15^, and found that *Chlorogloea fritschii* TISTR 8527 had a PHB accumulation and cell-cluster formation capability. This cyanobacterium exists in clump cell clusters (Fig. 1A) that spontaneously sedimented to the bottom of liquid medium within 1 min (Fig. 1B). In contrast, the unicellular cyanobacterium *Synechocystis* sp. PCC6803 does not have such a sedimentation ability (Fig. 1B). The self-sedimentation of *C. fritschii* enables the removal of the cell-free medium at the above portion of the sedimented cells leading to an easy harvest of the biomass. A biomass recovery of 91 ± 5% (w/w dry weight (DW)) was obtained, where 100% (w/w DW) was set as that recovered by centrifugation.

### Nitrate is required as a nitrogen source for rapid growth under photoautotrophy

*Chlorogloea fritschii* is a N_2_-fixing cyanobacterium. Thus, cell growth was assessed under the non-N_2_-fixing condition using the standard medium supplied with nitrate, and under the N_2_-fixing condition using the same medium without nitrate. The maximal biomass growth rate of 156 mg/L/d (during d8–d12) was obtained from the standard medium, while a 4.4-fold lower growth rate of 35 mg/L/d (during d8–d12) was derived from culture in the medium without nitrate (Fig. 1C). The time required to obtain the maximum biomass production (1595 mg/L) was 16 d in the standard medium, compared to 56 d (890 mg/L) in the medium without nitrate (Fig. 1C).

### Acetate is an efficient organic substrate for PHB accumulation

Five organic compounds, each chemically equivalent or similar to a metabolite in cyanobacterial carbon metabolism, were individually supplied to *C. fritschii* culture and evaluated for the PHB accumulation level (Table 1). Cells were cultured under the normal nutrient condition (NORMAL) or the nitrogen-limited condition (-N) under light. The -N culture yielded higher levels of PHB accumulation than the NORMAL cultures for both concentrations of all five organic substrates (Table 1). In the -N medium, acetate at 0.2 and 0.4% (w/v) yielded the highest PHB accumulation of 5.3 and 11.8% (w/w DW), respectively, after 20 d. Under the -N condition, acetate was deemed to be the most efficient organic substrate for PHB accumulation (Table 1) and was selected for subsequent experiments. Nevertheless, it is noted that under normal condition glucose and fructose at 0.4% (w/v) induced a 4-fold higher PHB accumulation than that of acetate at the same concentration.

### PHB accumulation is maximal under heterotrophy with nutrient deprivation in the dark

To increase PHB accumulation, the *C. fritschii* culture condition was optimized by adjusting the (i) amount of acetate supply, (ii) growth in the light or the dark and (iii) the nutrient supply as: normal nutrient condition (NORMAL), nitrogen limitation (-N), phosphorus limitation (-P), and nitrogen and phosphorus limitation (-N-P).

**Figure 2:**
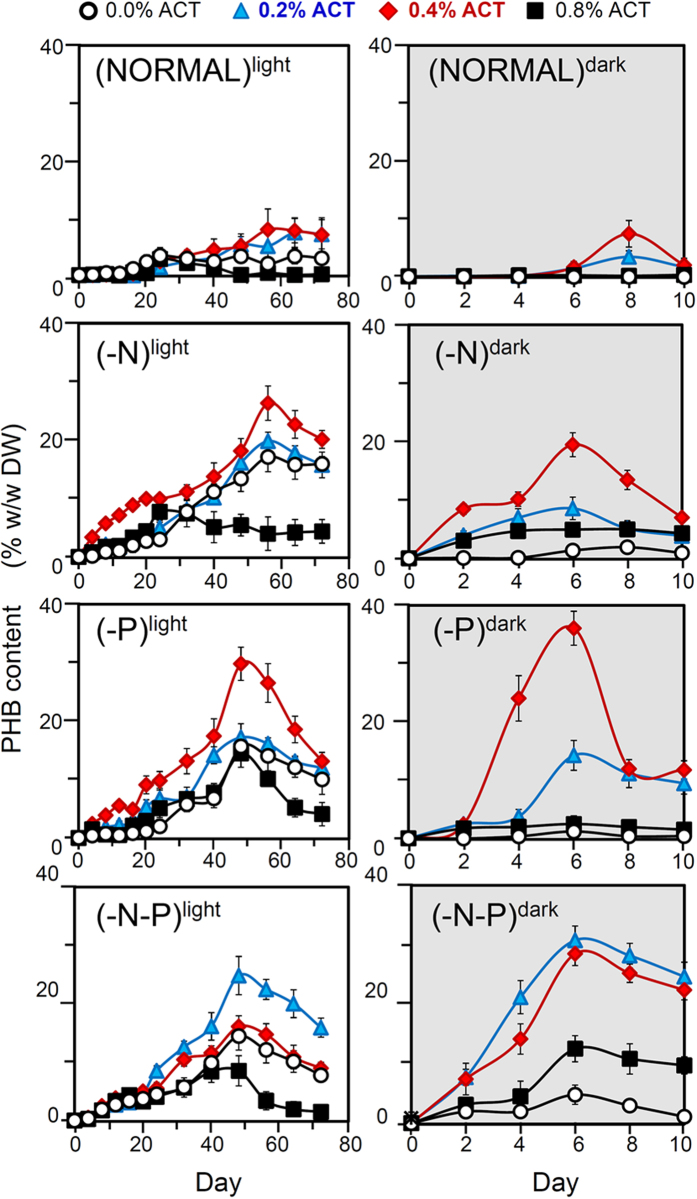
Cellular PHB contents under photoheterotrophy or heterotrophy in the dark with different initial levels of acetate (ACT) substrate. Cells were cultured under normal photoautotrophy for 16 d to increase their biomass and then transferred to the normal nutrient (NORMAL), or that deficient in nitrogen (-N), phosphorus (-P) or nitrogen and phosphorus (-N-P). Data are shown as the mean ± 1 SD, derived from three to six independent experiments.

Under photoautotrophy, the NORMAL condition yielded low PHB levels <4% (w/w DW), while culture in the -N, -P and -N-P media yielded moderate PHB levels at 14–17% (w/w DW) at the late period of the cultivation (≥48 d, Fig. 2).

Under mixotrophy (i.e. photoheterotrophy: acetate supply in the light), cultivation in the NORMAL medium yielded low PHB levels of <10% (w/w DW) at all acetate concentrations (Fig. 2). Of particular importance is that cultivation in the -N and -P media required acetate at 0.4% (w/v) to reach peak PHB levels at 26–30% (w/w DW). In contrast, the -N-P medium required only 0.2% (w/v) acetate to achieve the peak PHB level at 24% (w/w DW). All the peak PHB levels under this mixotrophy occurred in the late cultivation period (≥48 d, Fig. 2).

Under heterotrophy (acetate supply in the dark), cultivation in the NORMAL medium yielded a low PHB level of <10% (w/w DW) at all acetate concentrations (Fig. 2), while in the -N, -P and -N-P media a 0.2–0.4% (w/v) acetate concentration gave increased PHB levels to 8–30% (w/w DW) except at 0.4% (w/v) acetate in the -P medium where a higher PHB level of 36% (w/w DW) was obtained. Increasing the acetate concentration to 0.8% (w/v) reduced the PHB accumulation level compared to that at 0.2 and 0.4% (w/v) acetate. Interestingly, the -N and -P media required acetate at 0.4% (w/v) to reach peak PHB levels of 19–36% (w/w DW), while cultivation in the -N-P medium needed a 2-fold lower acetate concentration at 0.2% (w/v) to reach the peak PHB level of 30% (w/w DW). This result reflected the more efficient conversion of acetate to PHB under the -N-P condition.

Overall results in Fig. 2 indicate that although the PHB levels were not much different in cells under photoheterotrophy and under heterotrophy in the dark, the significant shortening of the cultivation time to achieve maximum PHB level was obtained under heterotrophy in the dark.

### Two-stage culture (photoautotrophy followed by heterotrophy in the dark) increased the PHB production

Next, *C. fritschii* was grown using the two-stage cultivation strategy. For the first stage, the cells were pre-grown under normal photoautotrophy for 16 d to achieve the maximum biomass level, whereby a PHB level of <1% w/w DW was detected in these 16-d pre-grown cells. Then, for the second stage, the cells were subjected to growth under the previously determined optimal heterotrophic condition (acetate as the substrate in the dark) to increase the PHB production. At this heterotrophic stage, the effects of different nutrient conditions (-N, -P or -N-P) and various acetate concentrations (0.2–0.8% w/v) on PHB levels were evaluated. In general, a decrease of acetate supplied to heterotrophic culture in the dark led to a decrease of PHB production in all nutrient deficient conditions, except at 0.4% (w/v) acetate under –N-P where PHB level appeared to saturate at 0.2% (w/v) acetate (Table 2). Of particular importance, heterotrophic culture in the -N and the -P medium required 0.4% (w/v) acetate to achieve the peak PHB production level of 267 and 531 mg/L, respectively, while in the -N-P medium a half-fold lower acetate concentration of 0.2% (w/v) gave the peak PHB production of 431 mg/L (Table 2).

With respect to the PHB yield of the two stage cultures, measured in terms of the % (w/w) [PHB product/acetate supply to the medium], the PHB yield was <14% (w/w) when cultured in the NORMAL, -N and -P media in the heterotrophic stage, compared to 9–29% (w/w) when cultured in the -N-P medium depending upon the acetate concentration (Table 2). The PHB yield was highest at 29% (w/w) when cultured in the -N-P medium with 0.1% (w/v) acetate (Table 2).

### Conversion efficiency (*CE*) of acetate to PHB

**Figure 3:**
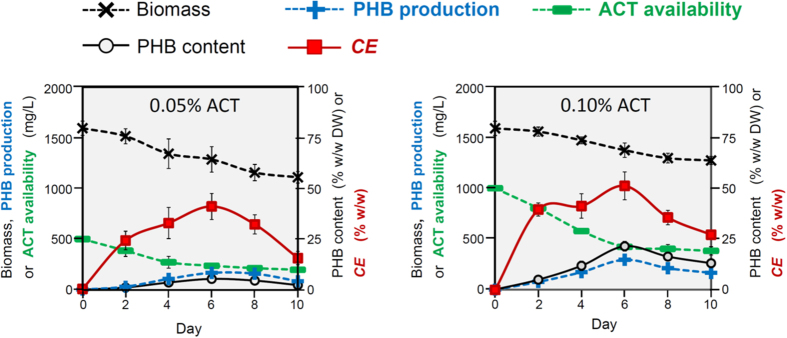
Conversion efficiency (*CE*) of acetate to PHB in the two-stage cultures of *C. fritschii*. Sixteen-d photoautotrophy-grown cells were transferred to the heterotrophic -N-P medium in the dark up to 10 d with an initial acetate (ACT) concentration of 0.05% or 0.1% (w/v). The residual available ACT level in the culture medium and *CE* of acetate to PHB was determined at the indicated time points. Data are the average ± 1 SD from six independent cultures.

The mass conversion efficiency of acetate to PHB (*CE*^*Acetate→PHB*^) was determined in two-stage cultures in terms of the % (w/w) [PHB product/cellular acetate consumption]. The maximal *CE*^*Acetate→PHB*^ of 51% was obtained from the two-stage culture in which the cells during heterotrophic stage were grown in the -N-P medium with 0.1% (w/v) acetate in the dark (Fig. 3). Reducing and increasing the acetate concentration to 0.05% and 0.2% (w/v) under the same condition decreased the *CE*^*Acetate→PHB*^ to 41% (Fig. 3) and 33% (data not shown), respectively.

### Acetyl-CoA level and PHB synthase activity

The acetyl-CoA level and PHB synthase activity of the cells cultured in the different heterotrophic media (NORMAL, -N, -P or -N-P) with added acetate at 0.1 and 0.2% (w/v) of the two-stage cultures were determined. Cells cultured in the -N medium had a two-fold increased PHB synthase activity compared to those in the NORMAL medium, while in the -P or -N-P medium it was six- to eight-fold higher (Table 3). Consistent with this, was that in the -N medium the cells had a slightly (1.2- to 1.3-fold) raised acetyl-CoA level (presumably, the primary substrate of PHB synthesis in *C. fritschii*), while in the -P and -N-P media the cells had a much higher level of acetyl-CoA by 2.0- to 2.8-fold, relative to those in the NORMAL medium (Table 3). The increase of added acetate from 0.1 to 0.2% (w/v) had little effect on both the enzyme activity and the acetyl-CoA levels. Overall, the results indicate that heterotrophically grown *C. fritschii* cells in -P or -N-P medium, rather than -N medium, had considerable increase of both the precursor and the enzyme activity required for PHB production.

### Material properties of PHB from *C. fritschii*

The PHB extracted from *C. fritschii* cultured under the two-stage culture was examined by ^13^C- and ^1^H-NMR spectra, and was found to match the respective spectra of the commercial PHB (Supplementary Fig. S1). The thermal and physical properties of *C. fritschii* PHB were comparable to those of the commercial PHB (Table 4), except for a slightly lower enthalpy of fusion (*ΔH*_*m*_) and % crystallinity (*X*_*c*_) observed in *C. fritschii* PHB. Interestingly, *C. fritschii* PHB had a 27% decreased polydispersity (*M*_*w*_*/M*_*n*_) and a 22–43% decreased molecular weight (*M*_*w*_ and *M*_*n*_) relative to the commercial PHB (Table 4).

## Discussion

Microbial production of PHB and none-excreted biomolecules requires the separation of the microbial biomass from the liquid culture medium. Centrifugation and chemical flocculation have previously been used to recover the biomass^16^,^17^, but these methods are extensively energy, time and cost consuming. In the filamentous cyanobacterium *Arthrospira platensis*, filtration has been used for cell harvesting, but the method requires considerable amount of time and cost for filtration equipment^18^. Recently, *Anabaena* sp. producing H_2_ gas has been shown to float on top of the culture medium enabling its ease of biomass recovery^19^. However, this requires the restrictive anaerobic culturing system for H_2_ production^19^. Here, we demonstrated that the cell-cluster forming *C. fritschii* exhibited spontaneous sedimentation (Fig. 1B). This enables the removal of the cell-free medium at the above portion of the sedimented cells (Fig. 1B), facilitating an easy harvesting of the biomass at a 91 ± 5% (w/w) recovery. The self-sedimentation feature of *C. fritschii* was found in all phases of photoautotrophic growth, and the larger size of cell clusters were found at the late log phase and the stationary phase (not shown). How *C. fritschii* exhibited high mass density and sedimented to the bottom of the culture is still unknown. This cell clustering feature has been observed in other cyanobacteria^20^,^21^, and so it is worth examining the auto-sedimentation ability in these strains.

Culturing in the -N or –P medium under the heterotrophic condition was found to increase the PHB accumulation in *C. fritschii* (Fig. 2), consistent with previous reports in other cyanobacteria under photoautotrophy^1^,^2^,^15^ and/or heterotrophy^1^,^2^. Recently, the *Synechocystis* sp. PCC6803 photoheterotrophically grown with glucose was shown to have the highest PHB accumulation level in -N-P medium, about a 1.4- to 1.6-fold greater than that in the -N or -P medium^3^. Likewise, this study showed a 2- to 18-fold greater PHB accumulation when *C. fritschii* was cultured in the -N-P medium, than in the -N or -P medium under the heterotrophy with acetate supply at ≤0.2% (w/v) in the dark (Table 2). This greater PHB storage when cultured in the -N-P medium than that in the -N medium is attributed to the higher levels of acetyl-CoA and the superior PHB synthase activity found in cells cultured in the -N-P medium (Table 3). However, it is noted that broadly similar acetyl-CoA and PHB synthase activity levels were found in the -P cultures and in the -N-P cultures (Table 3), whereas the latter had a higher PHB accumulation level at almost all acetate concentrations (Table 2). This might be due to the increased levels of other enzymes and metabolites in the *C. fritschii* PHB synthetic pathway that have not been identified at present. In the cyanobacterium *Synechocystis* sp. PCC6803, where the PHB synthesis pathway has been completely identified, culturing in -N or -P medium was found to enhance the photoautotrophic PHB accumulation by increasing the activity levels of the three enzymes in the PHB synthetic pathway^22^,^23^,^24^.

In this study, the maximal PHB production by *C. fritschii* at 531 mg/L after 22 d of cultivation was obtained under the two-stage cultivation system when the heterotrophic stage was performed in the -P medium with 0.4% (w/v) acetate in the dark (Table 2). A higher cyanobacterial PHB production level of 1,597 mg/L after 19 d was previously reported in *Aulosira fertilissima* cultured heterotrophically in the dark with multiple types of organic substrates^25^. However, the *CE* of these substrates to PHB *by A. fertilissima* was not determined.

Based on the biochemical pathway, the maximal theoretical mass *CE*^*Glucose→PHB*^ and *CE*^*Acetate→PHB*^ is 48% (w/w)^7^. Bacterial *CE*^*Acetate→PHB*^ and *CE*^*Glucose→PHB*^ levels of 26% and 36–42% (w/w), respectively, have been described previously, but these bacteria also required other composite organic substrates, such as yeast extract and/or peptone, in the media for PHB production^8^,^9^,^26^. The *CE*^*Glucose→PHB*^ above the theoretical value at 52% (w/w) was reported in the bacterium *Halomonas* TD01, but again this bacterium required yeast extract as a composite organic substrate for the PHB production^10^. The definitive determination of the *CE* from a specific single organic substrate to PHB requires a culture system that contains only the organic substrate of interest, as was performed in this work. Here, *C. fritschii* was pre-cultured under photoautotrophy that yielded high biomass level but low PHB accumulation (<1% w/w DW), and followed by heterotrophic culture with acetate as the only organic substrate to promote PHB production. In this two-stage culture a maximal *CE*^*Acetate→PHB*^ of 51 ± 7% (w/w) was obtained (Fig. 3). This obtained *CE*^*Acetate→PHB*^ is comparable to the theoretical biochemical efficiency of 48% (w/w)^7^. That this conversion is comparable to the theoretical efficiency may be attributed to the fact that *C. fritschii* was pre-grown under photoautotrophy, where cells would store a certain amount of energy and metabolites in their biomass and convert particular molecules to PHB during the subsequent heterotrophic culture. In support of this contention, a substantial biomass reduction (20 and 14% under 0.05 and 0.10% w/v acetate, respectively) was found 6 d after transferring photoautotrophically grown *C. fritschii* to the heterotrophic cultures (Fig. 3). In the second-stage cultivation, how acetate was metabolized to PHB and whether certain metabolites were converted to PHB remain to be elucidated. The metabolic flux analysis using radioactively labeled acetate and the related metabolites would provide an insight into this cellular mechanism.

In conclusion, this work demonstrated the efficient bioconversion of acetate to PHB, by the easy-to-harvest cyanobacterium *C. fritschii*. The results demonstrated the mass *CE* of acetate to PHB is at the comparable level to the theoretical efficiency. Further metabolic engineering that targets carbon storage pathways, such as that recently described by Osanai *et al.*^22^,^23^, and shortening culture time by optimizing light intensity, temperature and carbon supply, as demonstrated by Yu *et al.*^27^, would help to increase the *CE*. The established algal co-culture technique^28^, might be applied for concurrent cyanobacterial cultivation between a PHB-producing strain and a photoautotrophically-grown acetate-excreting strain^29^ to produce the biopolymer directly from CO_2_ and solar energy without the need for an additional organic supply.

## Methods

### Strain and culture conditions

The axenic culture of *Chlorogloea fritschii* TISTR 8527 was obtained from the Thailand Institute of Scientific and Technological Research. The strain was previously isolated from a freshwater pond in Bangkok and purified by the alkaline and ampicillin treatment method^30^. An approximately 5% (v/v) of a 16-d old culture was inoculated into 250-mL flasks containing 150 mL BG-11 medium^31^ without sodium citrate and supplemented with 20 mM HEPES-NaOH (pH 7.5) and with ferric ammonium citrate replaced by ferric chloride. Thus, this BG11 medium does not contain any organic compounds. Cells were grown at 32 °C under a continuous white light of 100 μmol/m^2^/s using an atmospheric CO_2_ supply upon shaking at 160 rpm. The limitation of combined nitrogen source and/or phosphorus in the BG11 medium was proceeded by making the medium devoid of such nutrients, as previously described^3^. Sodium acetate, sodium pyruvate, sodium citrate, glucose or fructose (Sigma-Aldrich, St. Louis, MO, USA) was added to the BG11 medium when required. The dark condition was acquired by wrapping each culture flask in an aluminum sheet. Wet cells were immediately frozen by liquid nitrogen and dried at 65 °C.

### Quantification of PHB

The PHB content, as % (w/w DW), was determined using high-performance liquid chromatography (HPLC) as described^32^. Dry cells were boiled in H_2_SO_4_ to hydrolyze the PHB into crotonic acid. The crotonic acid content was then analyzed by HPLC using adipic acid as the HPLC marker. Commercial PHB (Sigma-Aldrich, St. Louis, MO, USA) was analyzed in parallel, where an 84.6 ± 4.0% (w/w) conversion of PHB to crotonic acid was obtained.

### Quantification of acetate in culture medium

The acetate level in the medium was quantified using the HPLC system for organic acid analysis according to the protocol of Shimadzu (Japan). The HPLC was equipped with an InertSustain Carbon-18 column and 10 mM NH_4_H_2_PO_4_ (adjusted to pH 2.6 by H_3_PO_4_) as the mobile phase. The UV detection at 210 nm was used to detect acetate, while BG11 medium containing 0.1–2.0 g/L sodium acetate was used as quantification standards.

### Determination of the *CE* of acetate to PHB

The *CE* proceeded by the cells was calculated according to Eq. (1),


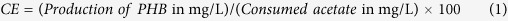


where *Production of PHB* is the difference in production at the second-stage heterotrophic culture and at the end of the first-stage photoautotrophic culture, *Consumed acetate* is the difference in acetate concentration in the medium at the initial time of the cultivation and at the specific time point after starting the cultivation.

### Assay of PHB synthase activity and acetyl-CoA level

The PHB synthase activity was determined essentially as previously described^33^. Each 1-ml reaction contained 0.2 mg of crude protein, 1.5 mM 3-hydroxybutyryl-CoA substrate and 0.5 mM 5,5-dithiobis(2-nitrobenzoic acid) in Tris-glycerol buffer (25 mM Tris-HCl pH 7.5, containing 5% (v/v) glycerol). The reaction was initiated by adding 3-hydroxybutyryl-CoA and incubated at 30 °C for 10 min. The resulting thiobenzoate anion was measured spectrophotometrically at 412 nm. Protein quantification was performed using the Lowry method.

The acetyl-CoA level was determined using the acetyl-CoA assay kit MAK039 (Sigma-Aldrich, St. Louis, MO, USA). Cells were harvested and frozen in liquid nitrogen. Acetyl-CoA was specifically catabolized by the enzyme assay kit according to the manufacturer’s protocol and the resulting product was quantified by its fluorescent intensity (535-nm excitation and 587-nm emission).

### Extraction of PHB and analysis of its properties

For PHB extraction, dry cells were washed in methanol to remove pigments and then the PHB was extracted in chloroform followed by precipitation using diethyl ether^34^. For nuclear magnetic resonance (NMR), 2 mg/mL PHB in deuterochloroform (CDCl_3_) was analyzed by ^1^H- and ^13^C-NMR at 25 °C using a Bruker Advance 400 MHz spectrometer (Germany). For thermal properties, 15 mg of PHB was analyzed by differential scanning calorimetry (Netzsch DSC-204-F1 machine, Germany) in the range from −50 °C to 200 °C. For mechanical analysis, the PHB film was cut into rectangles and analyzed at 25 °C using Hounsfield H10KM material testing machine (UK). For molecular weight determination, 2 mg/mL PHB in chloroform was analyzed by gel permeation chromatography (Shimadzu, Japan) using the K802.5, K803 and K804 columns (Shodex, NY, USA) according to the manufacturer’s method. Polystyrene in the range of 4.0 × 10^2^–1.8 × 10^6^ g/mol (Sigma-Aldrich, St. Louis, MO, USA) was analyzed as the mass standards.

## Additional Information

**How to cite this article**: Monshupanee, T. *et al.* Two-stage (photoautotrophy and heterotrophy) cultivation enables efficient production of bioplastic poly-3-hydroxybutyrate in auto-sedimenting cyanobacterium. *Sci. Rep.* **6**, 37121; doi: 10.1038/srep37121 (2016).

**Publisher’s note:** Springer Nature remains neutral with regard to jurisdictional claims in published maps and institutional affiliations.

## Supplementary Material

Supplementary Information

## Figures and Tables

**Table 1:** PHB contents under photoheterotrophy supplied with a different organic substrate.

Organic substrate	PHB content (% w/w DW)			
	NORMAL		-N	
	Supplied concentration (% w/v)			
	0.2	0.4	0.2	0.4
Acetate	0.7 ± 0.2*	0.8 ± 0.4*	5.3 ± 1.2*	11.8 ± 2.1*
Pyruvate	0.3 ± 0.2	0.4 ± 0.3	0.7 ± 0.7	0.5 ± 0.4
Citrate	0.4 ± 0.2	0.3 ± 0.1	4.2 ± 0.3*	5.7 ± 0.9*
Glucose	0.7 ± 0.3*	3.4 ± 1.0*	3.1 ± 5.3*	6.7 ± 0.4*
Fructose	1.2 ± 0.5*	3.2 ± 0.9*	2.8 ± 1.2*	8.1 ± 1.4*

5% (v/v) of *C. fritschii* was cultured in normal nutrient (NORMAL) or nitrogen-limiting (-N) medium containing the indicated organic substrate under light for 20 d.

Data are the average ± 1 SD from three independent cultures.

^*^Significantly higher PHB content (two-tailed *t-*test, *P* < 0.05.) than the PHB content obtained from the same nutrient condition, but without organic substrate (NORMAL = 0.20 ± 0.12% w/w DW, -N = 1.42 ± 0.45% w/w DW).

**Table 2:** PHB production under a two-stage (photoheterotrophy, then heterotrophy) culture.

Nutrient condition	Acetate supply % (w/v)	PHB content (% w/w DW)	PHB production (mg/L)	% w/w [PHB product/ACT supply]^a^
NORMAL	0.00	0.1 ± 0.0	1 ± 1	na
	0.05	0.1 ± 0.0	2 ± 1	0.0 ± 0.0
	0.10	0.6 ± 0.4	9 ± 5	0.7 ± 0.5
	0.20	1.5 ± 0.9	22 ± 13	0.1 ± 0.6
	0.40	1.8 ± 0.8	28 ± 15	0.7 ± 0.3
-N	0.00	1.4 ± 0.8*	19 ± 10*	na
	0.05	0.4 ± 0.2*	6 ± 5	0.8 ± 0.9
	0.10	2.2 ± 0.9*	30 ± 11*	2.8 ± 1.0*
	0.20	8.6 ± 1.9*	121 ± 23*	6.0 ± 1.1*
	0.40	19.5 ± 2.0*	267 ± 16*	6.6 ± 0.4*
-P	0.00	1.3 ± 0.1*	18 ± 4*	na
	0.05	1.8 ± 1.0*	25 ± 17*	4.6 ± 3.3*
	0.10	7.5 ± 1.5*	97 ± 11*	9.5 ± 1.1*
	0.20	14.3 ± 2.5*	203 ± 30*	10.0 ± 1.5*
	0.40	36.0 ± 2.8*	531 ± 75*	13.2 ± 1.8*
-N-P	0.00	4.9 ± 1.6*	65 ± 24*	na
	0.05	8.3 ± 1.9*	108 ± 30*	21.2 ± 6.0*
	0.10	21.4 ± 2.4*	295 ± 37*	29.3 ± 3.7*
	0.20	30.7 ± 2.8*	431 ± 45*	21.5 ± 2.2*
	0.40	28.5 ± 1.9*	395 ± 9*	9.8 ± 0.2*

Cells were pre-grown under normal photoautotrophy for 16 d and transferred to the specified heterotrophic conditions using the 6-d time culture period yielding the highest PHB contents (Fig. 3). Data are the average ± 1 SD from three to five independent cultures. Asterisks indicate significantly higher levels (*P* < 0.01, two-tailed *t-*test) than those obtained from NORMAL nutrient condition and the same acetate supply. na, not applicable. ^a^ = [(*PHB production, mg/L*)]/(*ACT supply in the culture medium, mg/L*)] × 100, where *PHB production* is the difference in production at a second-stage heterotrophic culture and at the end of the first-stage photoautotrophic culture.

**Table 3:** Cellular acetyl-CoA and PHB synthase activity levels after heterotrophic culture in the dark.

Nutrient condition	Acetyl-CoA level (μg/g DW)		PHB synthase activity (nmol/min/mg protein)	
NORMAL	25.2 ± 5.1	20.3 ± 6.1	15.2 ± 4.2	13.2 ± 4.1
-N	30.7 ± 11.1	27.5 ± 9.7	35.1 ± 8.5*	31.3 ± 5.5*
-P	56.5 ± 10.7*	58.4 ± 11.7*	105.3 ± 15.3*	97.4 ± 10.1*
-N-P	52.1 ± 13.5*	56.6 ± 12.5*	98.5 ± 19.4*	111.5 ± 12.3*

Cells were cultured as described in Table 2. Data are average ± 1 SD from five independent experiments. Asterisks indicate significantly higher levels (*P* < 0.01, two-tailed *t-*test) than that obtained from NORMAL nutrient condition with the same acetate supply.

**Table 4:** Material properties of PHB from *C. fritschii*.

Sources of PHB	Thermal properties					Mechanical properties^2^			Molecular weight		
	*T*_*m*_ (°C)	*T*_*g*_ (°C)	*T*_*cc*_ (°C)	*∆H*_*m*_ (J/g)	*X*_*c*_ (%)	Elongation at break (%)	Tensile strength (MPa)	Young’s modulus (MPa)	*M*_*w*_ (kDa)	*M*_*n*_ (kDa)	*M*_*w*_/*M*_*n*_
Commercial PHB^1^	175.4 (159)	3.5	48	99	68	5.8 ± 1.1	24 ± 3	820 ± 300	970	330	2.9
*Chlorogloea fritschii*	171.6 (171)	3.2	54	**65**	**45**	5.5 ± 1.8	23 ± 6	712 ± 256	**545**	256	**2.1**

Sixteen-d old photoautotrophically-grown cells were transferred to -N-P medium in the dark with 0.1% (w/v) acetate for 6 d. The dried biomass was extracted for PHB. *T*_*m*_, melting temperature (first melting peak shown in parentheses); *T*_*g*_, glass-transition temperature; *T*_*cc*_, cold-crystallization temperature; *∆H*_*m*_, enthalpy of fusion; *X*_*c*_, crystallinity; *Mw*, weight-average molecular weight; *Mn*, number-average molecular weight; *Mw/Mn*, polydispersity.

^1^Data from Sigma-Aldrich (St. Louis, MO, USA).

^2^The mechanical properties are shown as the mean ± 1 SD of three independent experiments.
