# Supplementary material for: Two-stage (photoautotrophy and heterotrophy) cultivation enables efficient production of bioplastic poly-3-hydroxybutyrate in auto-sedimenting cyanobacterium
Source: Sci Rep. 2016 Nov 15;6:37121. doi: 10.1038/srep37121 (PMC5109257; doi:10.1038/srep37121)
Supplement: Supplementary Information [file srep37121-s1.pdf]

Supplementary information

Two-stage (photoautotrophy and heterotrophy) cultivation enables efficient production of bioplastic poly-3-hydroxybutyrate in auto-sedimenting cyanobacterium

Tanakarn Monshupanee*, Palida Nimdach and Aran Incharoensakdi

Department of Biochemistry, Faculty of Science, Chulalongkorn University, Bangkok 10330, Thailand. **Correspondence:* tanakarn.m@chula.ac.th

Fig. S1. ^{1}H-NMR (upper) and ^{13}C-NMR (lower) spectra of the purified PHB from *C. fritschii*. Cells were cultured as described in Fig. 4. NMR peaks matching to each hydrogen and carbon atoms in the PHB chemical structure are marked by numerical numbers.

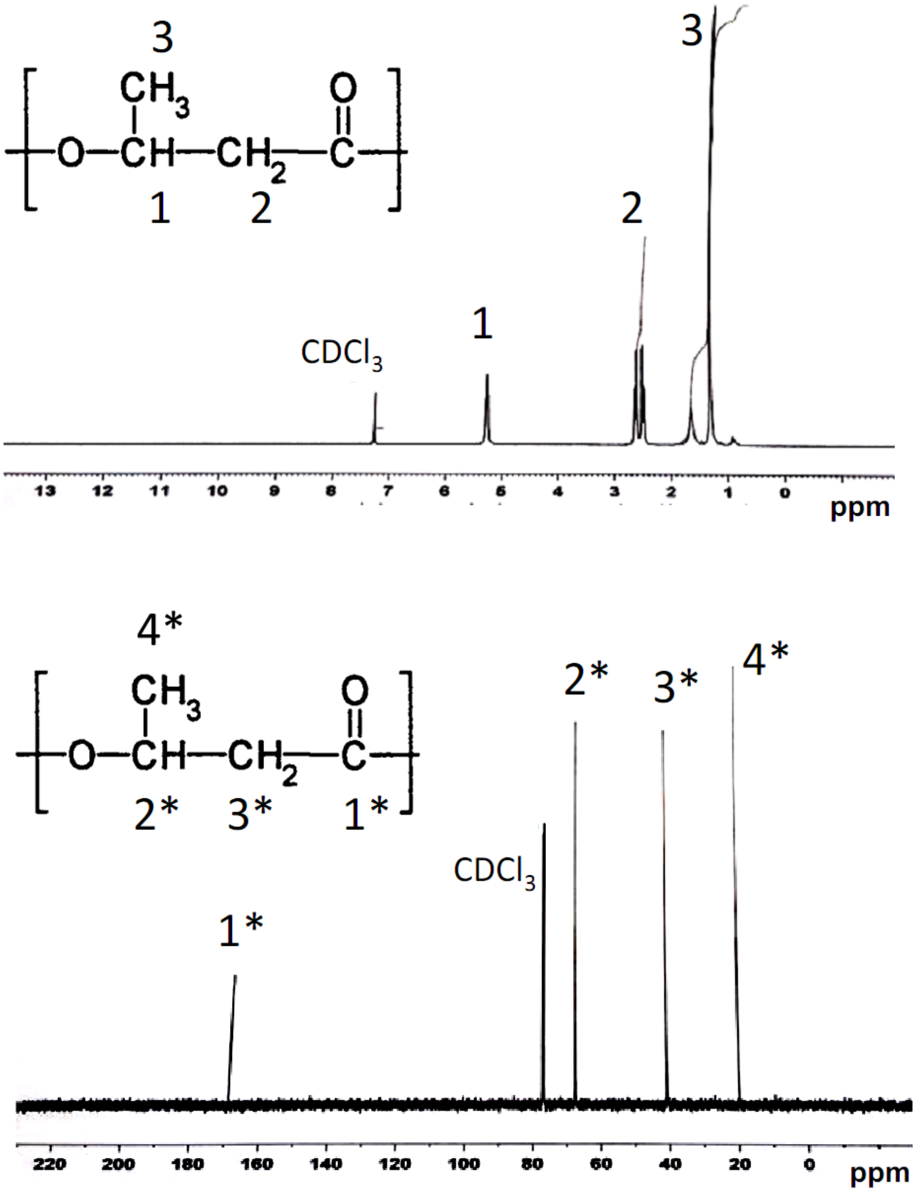

--- *end page* ---------------------------------------
